# Ionizing radiation-induced microRNA expression changes in cultured RGC-5 cells

**DOI:** 10.3892/mmr.2015.3938

**Published:** 2015-06-16

**Authors:** KAIJUN WANG, MEIJUAN ZHU, PANPAN YE, GUODI CHEN, WEI WANG, MIN CHEN

**Affiliations:** 1 Eye Center, Medical College of Zhejiang University, Hangzhou, Zhejiang 310009, P.R. China; 2 Zhejiang Provincial Key Laboratory of Ophthalmology, Medical College of Zhejiang University, Hangzhou, Zhejiang 310009, P.R. China; 3 Department of Radiation Oncology, The Second Affiliated Hospital, Medical College of Zhejiang University, Hangzhou, Zhejiang 310009, P.R. China

**Keywords:** ionizing radiation, microRNAs, retinal ganglion cell

## Abstract

MicroRNAs (miRNAs) are a class of short non-coding RNAs that regulate gene expression at the post-transcriptional level. It has been demonstrated that miRNAs serve a crucial role in tissue development and the pathogenesis of numerous diseases. The aim of the current study was to investigate the alterations in miRNA expression in a cultured retinal ganglion cell line (RGC-5 cells) following ionizing radiation injury. Cultured RGC-5 cells were exposed to X-rays at doses of 2, 4, 6 and 8 Gy using a medical linear accelerator. Alterations in cellular morphology were observed under a phase contrast microscope and cell viability was measured using the MTT assay. Subsequent to exposure to X-ray radiation for 5 days, the viability of RGC-5 cells was significantly reduced in the 6 and 8 Gy groups, accompanied by morphological alterations. Total RNA was then extracted from RGC-5 cells and subjected to miRNA microarray analysis subsequent to exposure to 6 Gy X-ray radiation for 5 days. The results of the microarray analysis indicated that the expression levels of 12 miRNAs were significantly different between the 6 Gy and control groups, including 6 upregulated miRNAs and 6 downregulated miRNAs. To verify microarray results, a reverse transcription-quantitative polymerase chain reaction (RT-qPCR) analysis was performed. The data obtained from RT-qPCR analysis was similar to that of the the microarray analysis for alterations in the expression of the 12 miRNAs. The results of the current study indicated that miRNA expression was sensitive to ionizing radiation, which may serve an important role in mechanisms of radiation injury in retinal ganglion cells.

## Introduction

MicroRNAs (miRNAs) are a class of short non-coding RNAs that regulate gene expression predominantly at the post-transcriptional level. At present, almost 2,000 unique mature human miRNAs have been identified, which serve critical roles in tissue development and the pathogenesis of numerous diseases (1–3). Greater than 250 miRNAs have been reported to be expressed in the retina (4). It has been demonstrated that miRNA-mediated gene regulation is a major mechanism underlying retinal and optic nerve development, function and associated diseases (5).

Radiation-induced retinopathy and radiation-induced optic neuropathy (RION) are severe complications of radiotherapy for intracranial, skull-base and sinus tumors, which may result in irreversible visual loss. Approximately 85% of affected eyes have final visual acuity of 6/60 or worse (6). However, the detailed molecular mechanisms of radiation-induced retinopathy and RION remain unclear. Previous studies have demonstrated that miRNA expression is sensitive to ionizing radiation injury and alterations in miRNA expression appear to be important in the mechanisms of radiation-induced injury and associated disease (7,8).

Although radiation-induced retinopathy and RION have been extensively investigated, the clinical therapeutic options remain limited (9). According to the characteristics of these diseases, early diagnosis is extremely important for prevention and treatment of RION (10,11). It has been reported that miRNAs can serve as improved biomarkers for the early diagnosis of numerous diseases, including cancer (12), autoimmune diseases (13), cardiovascular diseases (14), ectopic pregnancy (15) and systemic inflammatory response syndrome (16). In contrast to other biomarkers, miRNAs are protected from endogenous RNase activity and are highly stable. For this reason, they have been considered as ideal biomarkers for use in early diagnosis, prediction of prognosis and therapeutic management (17).

The current study aimed to screen for altered expression levels of miRNAs in RGC-5 cells exposed to ionizing radiation, in order to identify the possible mechanisms of these altered miRNAs in radiation injury, and to explore the possibility of using specific miRNAs as potential biomarkers for radiation-induced retinopathy and RION.

## Materials and methods

### RGC-5 cell culture

The RGC-5 cell line (American Type Culture Collection, Manassas, VA, USA) was grown in modified RPMI 1640 medium (Gibco Life Technologies, Carlsbad, CA, USA), containing 10% fetal bovine serum (Gibco Life Technologies) and 1% penicillin-streptomycin (100 U/ml penicillin and 100 *μ*g/ml streptomycin; Sigma-Aldrich, St. Louis, MO, USA) at 37°C and 5% CO_2_. Cells were usually passaged at a ratio of 1:20. During cultivation, RGC-5 cells exhibited the same morphological phenotype in different generations. For viability assays, cells were plated onto positively charged 96-well plates. For microarray and reverse transcription-quantitative polymerase chain reaction (RT-qPCR) analysis, cells were harvested from 25 cm^2^ filter-capped cell culture flasks.

### Radiation treatment and cell viability assay

The Siemens Medical Linear Accelerator (6MV Oncor; Siemens, Munich, Germany) was used in the current study to mimic radiation injury *in vitro*. RGC-5 cells were irradiated with 250 cGy/min dose rate X-rays at final doses of 2, 4, 6 and 8 Gy. Unexposed cells served as controls. Following radiation, cells were cultured in a humidified incubator for 5 days and morphological alterations were observed daily using a phase-contrast microscope (Eclipse 90i; Nikon, Tokyo, Japan). Cell viability was evaluated by an MTT assay according to the manufacturer's instructions. Briefly, 20 *μ*l of 5 mg/ml MTT dye (Sigma-Aldrich) was added to each well and cells were incubated at 37°C for 4 h. Subsequently, the supernatant was removed and purple-colored precipitates of formazan were dissolved by gently shaking for 10 min in 150 *μ*l dimethyl sulfoxide (Sangon Biotech Co., Ltd., Shanghai, China). The microplate was read at 570 nm (Benchmark Microplate Reader; Bio-Rad Laboratories, Inc., Hercules, CA, USA). The optical density (A value) of each sample was measured and applied for cell viability calculation.

### RNA extraction and microarray assay

Total RNA of RGC-5 cells was extracted and purified using the mirVana™ miRNA Isolation kit (Ambion Life Technologies, Austin, TX, USA) according to the manufacturer's instructions, then were checked for a RIN number to inspect RNA integration by an Agilent Bioanalyzer 2100 (Agilent Technologies, Inc., Santa Clara, CA, USA). To analyze miRNA expression, total RNA samples (100 ng) containing miRNAs were labeled with cyanine 3-pCp (Agilent Technologies, Inc.) using the Agilent miRNAs Complete Labeling and Hyb kit (Agilent Technologies, Inc.). Slides were then hybridized for 20 h at 55°C using a hybridization system (G2535A; Agilent Technologies, Inc.). Subsequent to hybridization, the slides were washed in Agilent GE Wash Buffer 1 with Triton X-102, followed by Agilent GE Wash Buffer 2 with Triton X-102. All slides were immediately scanned using the Agilent Microarray Scanner and Feature Extraction software, version 10.7 (Agilent Technologies, Inc.) with the default settings. Raw data were normalized by the Quantile algorithm with Gene Spring Software, version 11.0 (Agilent Technologies, Inc.).

### RT-qPCR

The expression levels of miRNAs were confirmed with SYBR-based quantitative PCR (RT-qPCR). Briefly, a total of 1 *μ*g RNA from each sample was transcribed into cDNA using the PrimeScript RT reagent kit (GeneCopoeia, Inc., Guangzhou, China) according to the manufacturer's instructions. The RT product (10 *μ*l) was diluted with H_2_O up to 50 *μ*l. cDNA was then amplified by PCR with primers specific to the target sequence (Table I). Amplification conditions were as follows: 60 min incubation at 37°C, followed by a 5 min termination reaction at 85°C. Dilutions of cDNA in the PCR were adjusted for each gene with the aim of remaining within the linear range of amplification. RT-qPCR was performed with SYBR Green Realtime PCR Master mix (GeneCopoeia, Inc.) according to the manufacturer's instructions. Cycling conditions were as follows: Initial denaturation for 10 min at 95°C, 40 cycles of melting (95°C for 20 sec), annealing (62.5°C for 20 sec) and extending (72°C for 20 sec). The relative alterations in expression for each miRNA were calculated by the cycle threshold method (ΔΔCt method) as described previously (17).

### Statistical analysis

Statistical analysis was performed with SPSS software, version 11.3 (SPSS, Inc., Chicago, IL, USA). For all assays, experiments were performed a minimum of three times. Data are presented as the mean ± standard deviation and were evaluated by one-way analysis of variance and Student's t-test. P<0.05 was considered to indicate a statistically significant difference.

## Results

### Effect of radiation on RGC-5 cell morphology and viability

**Figure 1:**
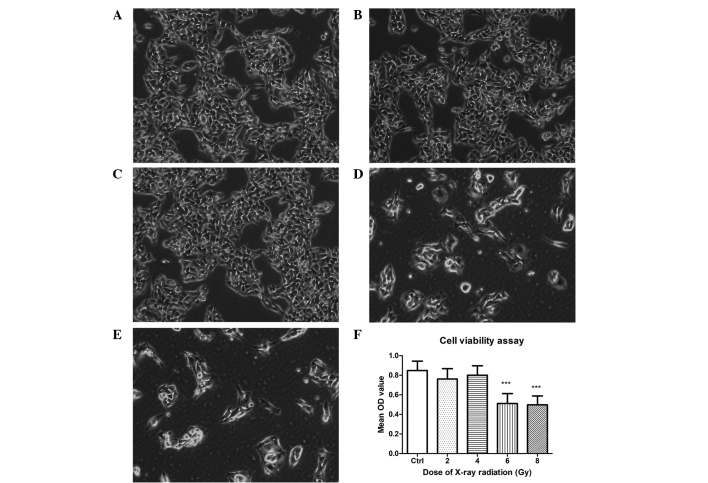
Morphological alterations and cell viability analysis of RGC-5 cells subsequent to exposure to different doses of radiation for 5 days, magnification ×100. (A) The control group. (B) Cells treated with 2 Gy radiation. (C) Cells treated with 4 Gy radiation. There were no significant alterations in the morphology of the cells in group B or C, compared with the control group. (D) 6 Gy radiation group. The density of cells significantly reduced compared with the control group. Rounding and swelling of RGC-5 cells was observed under the phase-contrast microscope. (E) 8 Gy radiation group. The density and shape of cells were similar to the 6 Gy group. (F) An MTT assay demonstrated that the cell viability significantly reduced in 6 Gy and 8 Gy groups compared with the control group (^***^P<0.01, n=4). There was no statistical significance between 6 Gy and 8 Gy groups (P>0.05). Cell viability of 2 Gy and 4 Gy groups did not change compared with the control group (P>0.05, one-way analysis of variance). OD, optical density.

As demonstrated by phase contrast microscopy, the majority of cells exhibited abnormal appearance in the 6 and 8 Gy groups following 5 days exposure to X-ray radiation, with the densities reduced and the cell shapes changing from a normal appearance to rounded and swollen. Compared with the control group, no significant morphological alterations were observed in the 2 and 4 Gy groups (Fig. 1A–E).

The MTT assay (Fig. 1F) indicated that the cell viability was significantly reduced in the 6 and 8 Gy groups following 5 days of radiation treatment (P<0.01). However, there was no significance difference between the 6 and 8 Gy groups (P>0.05). No significant differences in cell viability of the 2 and 4 Gy groups were identified compared with the control group (P>0.05), which was consistent with the morphological alterations described above.

### Alterations in miRNA expression following radiation injury

**Figure 2:**
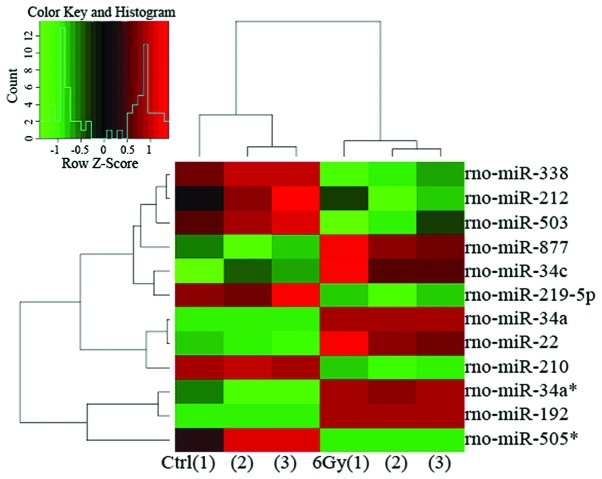
Heatmap of the 12 significantly altered miRNAs subsequent to exposure to 6 Gy radiation for 5 days. These 12 miRNAs were selected according to the fold changes and microarray repeatability. The upregulated miRNAs are depicted in shades of red and downregulated miRNAs are depicted in shades of green. The black blocks represent unchanged expression of the miRNAs. There were three samples (n=3) in each condition.

According to the results of the MTT assay, the 6 Gy dose of radiation was applied in RGC-5 cells to evaluate miRNA expression. In total, 677 miRNA probes were included in the miRNA microarray. Subsequent to exposure to 6 Gy radiation for 5 days, the expression levels of 37 miRNAs were statistically altered compared with the control group (P<0.05), with 16 upregulated miRNAs and 21 downregulated miRNAs. Among these altered miRNAs, 12 were selected according to the fold change (>1.5 or <0.667) and microarray repeatability (stable expression in triplicate experiments) (Fig. 2; repeated 3 times). Of these 12 miRNAs, 6 were upregulated [miRNA-192 (miR-192), -34a^*^, -877, -34c, -34a and -22; P<0.05 and fold change >1.5] and 6 were downregulated (miR-212, -338, -219-5p, -503, -210 and -505^*^; P<0.05 and fold change <0.667) (Table II).

### Independent RT-qPCR analysis to confirm microarray results

In order to validate microarray data, RT-qPCR analysis of miRNA expression was further performed for all of the 12 significantly altered miRNAs. The detailed RT-qPCR results compared with the results of microarray analysis (described as fold change) are presented in Table II. The expression levels of the 12 miRNAs exhibited similar alterations when measured by RT-qPCR (P<0.05), consistent with the results of microarray analysis.

## Discussion

miRNAs have been extensively investigated and have been recognized to act as critical regulators of gene expression by binding to their targets. miRNAs have revolutionized the understanding of gene regulatory networks, providing novel tools to manage the development and clinical therapy of numerous diseases. It has been demonstrated that miRNAs are essential mediators and serve key roles in retinal ganglion cell axon branching and optic nerve formation (18,19). However, the alterations in miRNA expression involved in radiation injury to RGCs and their detailed functions remain to be fully elucidated. In the current study, different doses of ionizing radiation were applied to induce radiation injury in cultured RGC-5 cells. Alterations in the miRNA expression levels were evaluated following radiation injury, which may aid in understanding the molecular mechanisms of radiation-induced retinopathy and RION.

The data of the present study revealed that exposure to 6 Gy and 8 Gy doses of X-ray radiation resulted in radiation injury in RGC-5 cells. The viability of RGC-5 cells was significantly reduced, which was in accordance with previously reported results (20,21). The microarray assay demonstrated that there was a total of 37 miRNAs that were statistically altered in the radiation-treated RGC-5 cells. It is known that when using microarray to detect differentially expressed miRNA, multiple tests are required to ensure that the results are stable and repeatable (22). A minimum of 3 repeats of each experiment were performed in the current study, following which 12 significantly altered miRNAs were selected according to the fold change and microarray repeatability, including 6 upregulated miRNAs (miR-192, -34a^*^, -877, -34c, -34a and -22) and 6 downregulated miRNAs (miR-212, -338, -219-5p, -503, -210 and -505^*^). Microarray technology is a powerful tool used to detect whether a set of miRNAs are differentially expressed. However, microarray profiling of microRNA expression raises a number of analytical challenges that must be addressed in order to obtain reliable results, and RT-qPCR analysis has been commonly used for further validation (23). In the current study, the data from RT-qPCR analysis were consistent with that from microarray analysis, indicating that the expression levels of these miRNAs were regulated in response to the radiation-induced injury in RGC-5 cells.

Ocular damage is a common side effect of radiotherapy, such as dry eye, epiphora, ectropion, scleral necrosis, cataract, glaucoma, optic neuropathy and retinopathy. Radiation-induced retinopathy and RION are the most serious complications in terms of visual damage resulting from radiation therapy for tumors adjacent to the retina and optic nerves (24). The pathogenesis of radiation-induced retinopathy and RION include vascular occlusion, injury to the retinal ganglion cells and axons or a combination of these effects (25). In a dog model of radiation-induced retinal injury, retinal degeneration with swelling and loss of the ganglion cells was observed following radiation treatment for 1 year (26). In humans, retinopathy commonly appears 6 months-3 years following radiation therapy, characterized by irreversible ganglion and glial cell necrosis and vasculitis (27). At present, the molecular mechanisms of ionizing radiation-induced ganglion cell injury remain to be fully elucidated. The data of the current study indicated that there were 12 miRNAs involved in the process of radiation-induced injury in RGC-5 cells. According to the reports, the function of these miRNAs and their regulated target genes are predominantly associated with the cell cycle and proliferation (miR-192, -22, -212 and -219-5p) (28–31), metabolism (miR-34a and -34c) (32) and apoptosis and anti-apoptosis (miR-192, -34a, -34c, -338 and -210) (28,33,34). For example, miR-192 is able to regulate p53 expression to induce tubular epithelial cell growth cycle arrest and DNA damage following kidney acute injury (28). miR-34a and miR-34c have been demonstrated to be downstream effectors of p53-mediated human fibroblast apoptosis and senescence (34). Further investigation of these miRNAs may aid in the understanding of the pathogenesis and development of radiation-induced retinopathy and RION.

Although numerous treatments have been suggested, management of radiation-induced retinopathy and RION remains controversial (9). At present, clinical therapeutic options include the use of corticosteroids, anticoagulants, antiaggregants and hyperbaric oxygen (HBO). A retrospective literature review identified there was no favorable effect of either corticosteroids or anticoagulation (35) and cases of radiation damage and visual loss have been reported to develop despite anticoagulation therapies (36). A previous study reported that HBO was a beneficial therapy for patients with radiation-induced retinopathy and RION (37). However, it has been suggested that HBO therapy is only effective in certain cases when patients were treated with HBO as early as possible following the onset of visual loss (37). According to characteristics of radiation-induced retinopathy and RION, the onset of visual acuity loss may be as short as 3 months or as long as 8 years following radiation exposure (38). Once radiotherapy has induced retinal complications, visual damage has been reported to progress quickly and be difficult to control (9). Early diagnosis is extremely important for the prevention and treatment of radiation-induced retinopathy (10,11). However, an effective method to monitor the early stages of radiation-induced retinopathy and RION remains to be developed.

A previous study demonstrated that miRNA expression is significantly different between pathological tissues and normal tissues (39). Understanding of the potential use of miRNA expression levels as biomarkers for the early detection of numerous diseases is on the increase (40). In the current study, 12 miRNAs, which were all sensitive to ionizing radiation and were differentially expressed between the experimental and control groups, were screened using microarray analysis in RGC-5 cells following radiation exposure. Due to the fact that it remains a challenge to detect and treat radiation-induced retinopathy and RION at an early stage, these 12 miRNAs are suggested as novel potential biomarkers for diagnosis, prediction of prognosis and therapeutic management.

Although the results of the current study are promising, there are several limitations. Firstly, a clear drawback of the present study is that it is an *in vitro* experiment. The environment of cells *in vivo* is different to cells cultured *in vitro*, however, it is easy to control the experimental conditions using cultured cells and the results are often more accurate. In addition, it has been identified that the majority of cells release pathogenic miRNAs into the extracellular environment of cells, where they are protected by RNase and remain relatively stable (41). Expression levels of miRNAs associated with diseases in biological fluids have been suggested as promising biomarkers for diagnosis and therapeutic monitoring (12–15). Concerning the 12 miRNAs screened in the current study, it remains unclear whether they are released by the retinal ganglion cells into the extracellular environment following radiation exposure. Thus, further studies are required to investigate the possibilities of the 12 miRNAs as potential biomarkers for radiation-induced retinopathy and RION.

In conclusion, the current study suggested that cultured RGC-5 cells were sensitive to ionizing radiation injury. The cell viability was significantly reduced following radiation exposure, accompanied by alterations in miRNA expression. These altered miRNAs may serve an important role in the mechanisms of radiation-induced retinopathy and RION, and may be used as potential biomarkers for the early detection of retinal and optic nerve radiation injury.

## Figures and Tables

**Table I:** Primer sequences used for the miRNA RT-qPCR analysis.

miRNAs	Primer sequence (5′–3′)
U6	AAGGATGACACGCAAATTCG
rno-miR-192	CTGACCTATGAATTGACAGCC
rno-miR-210	CTGTGCGTGTGACAGCGGCTGA
rno-miR-212	TAACAGTCTCCAGTCACGGCCA
rno-miR-219-5p	TGATTGTCCAAACGCAATTCT
rno-miR-22	AAGCTGCCAGTTGAAGAACTGT
rno-miR-338	TCCAGCATCAGTGATTTTGTTGA
rno-miR-34a	TGGCAGTGTCTTAGCTGGTTGT
rno-miR-34a^*^	AATCAGCAAGTATACTGCCCTA
rno-miR-34c	AGGCAGTGTAGTTAGCTGATTGC
rno-miR-503	TAGCAGCGGGAACAGTACTGCAG
rno-miR-505^*^	GGGAGCCAGGAAGTATTGATGTT
rno-miR-877	GTAGAGGAGATGGCGCAGGG

RT-qPCR, reverse transcription-quantitative polymerase chain reaction; miR, microRNA.

**Table II:** Microarray and RT-qPCR results of miRNA expression in RGC-5 cells subsequent to exposure to 6 Gy radiation for 5 days (fold changes).

miRNA name	Microarray	RT-qPCR
Upregulated		
miR-192	72.75	1.76^b^
miR-34a^*^	46.13	2.10^b^
miR-877	6.97	1.68^b^
miR-34c	2.71	1.70^b^
miR-34a	1.84	2.26^b^
miR-22	1.82	1.96^b^
Downregulated		
miR-212	0.66	0.38^a^
miR-338	0.64	0.48^b^
miR-219-5p	0.50	0.41^a^
miR-503	0.38	0.65^b^
miR-210	0.28	0.58^b^
miR-505^*^	0.02	0.26^b^

a P<0.05,

b P<0.01, fold change of microarray vs. RT-qPCR, (Student's t-test, n=3). RT-qPCR, reverse transcription-quantitative polymerase chain reaction; miRNA, microRNA.
